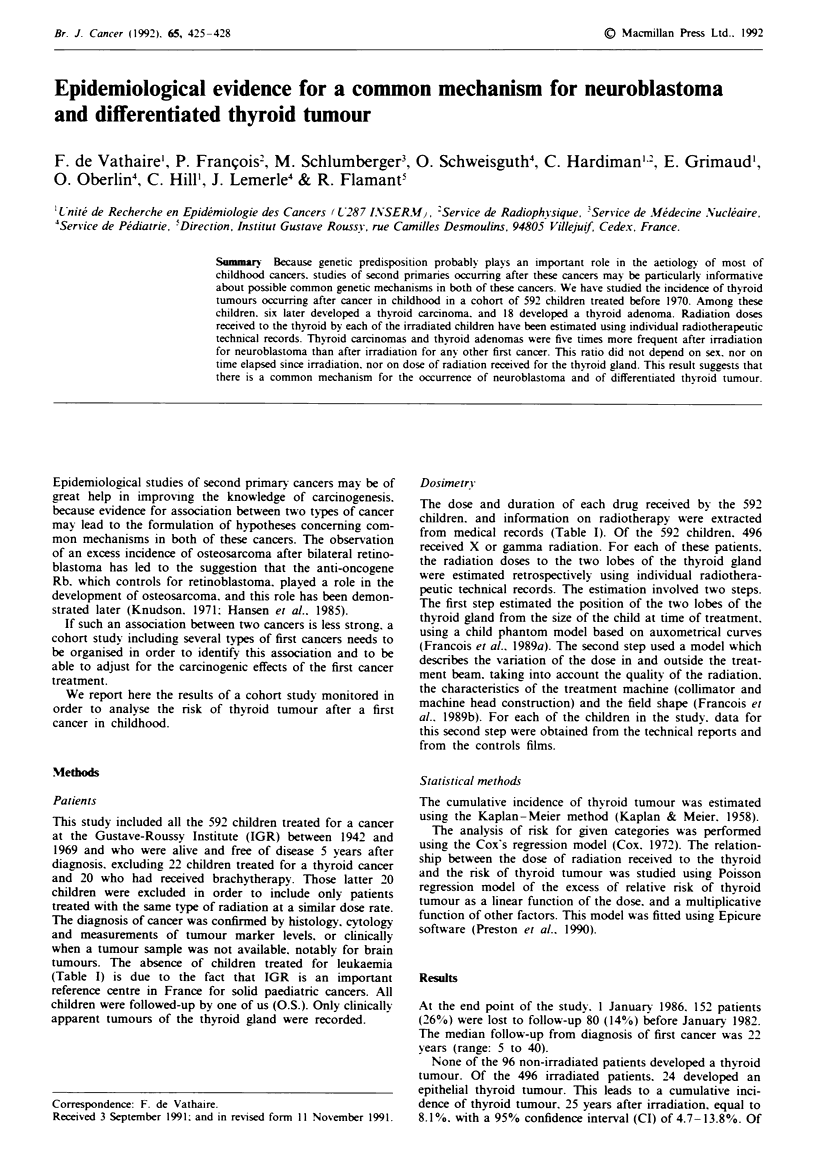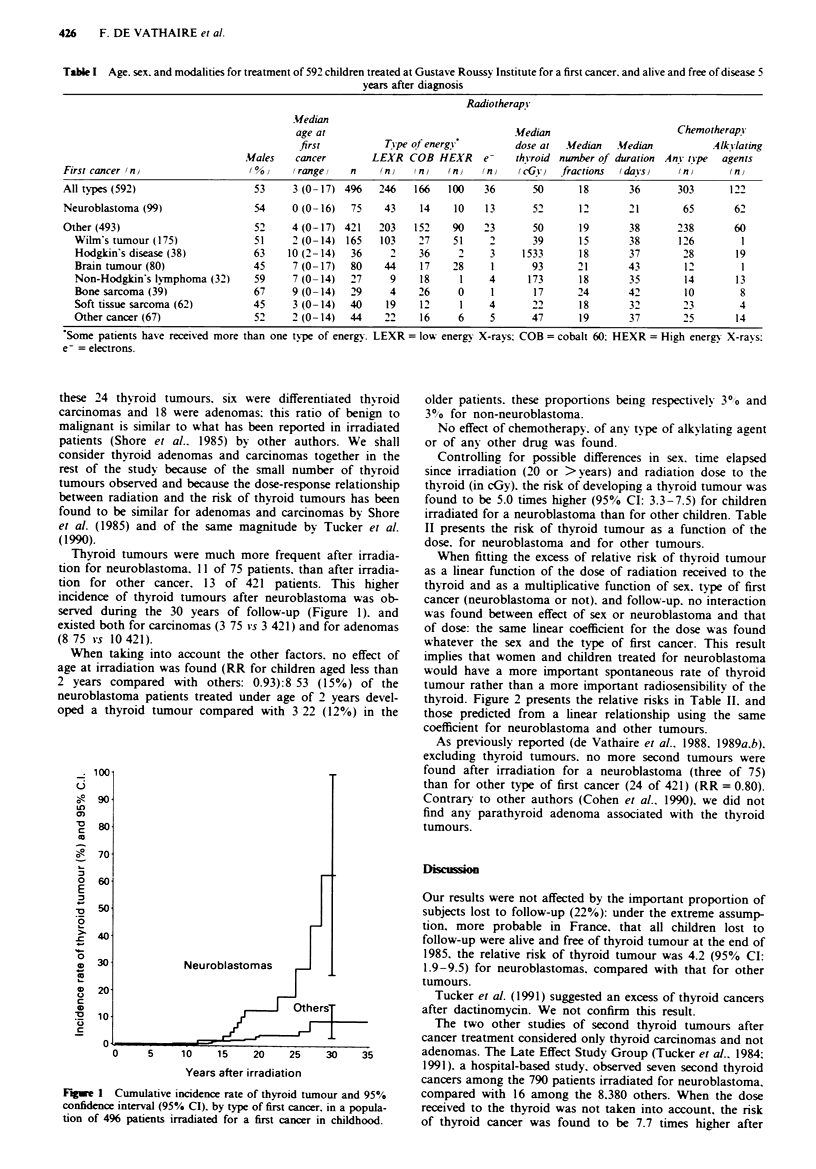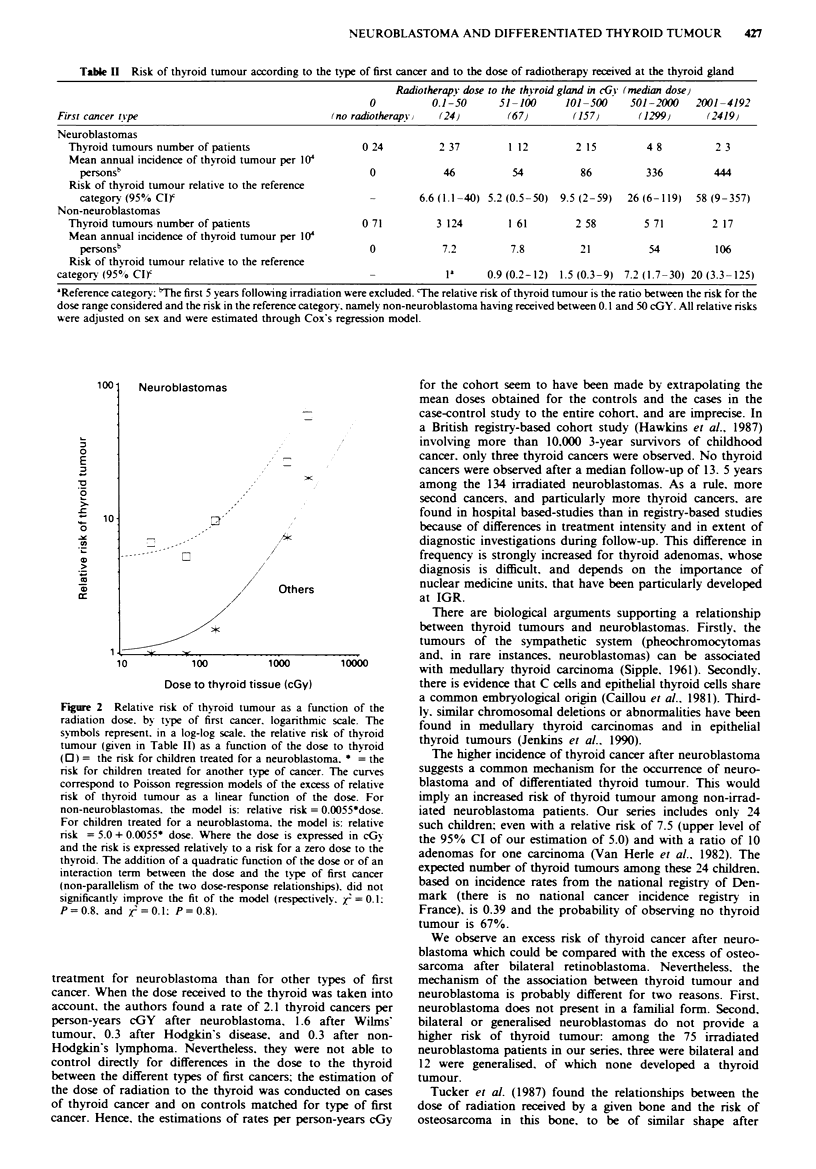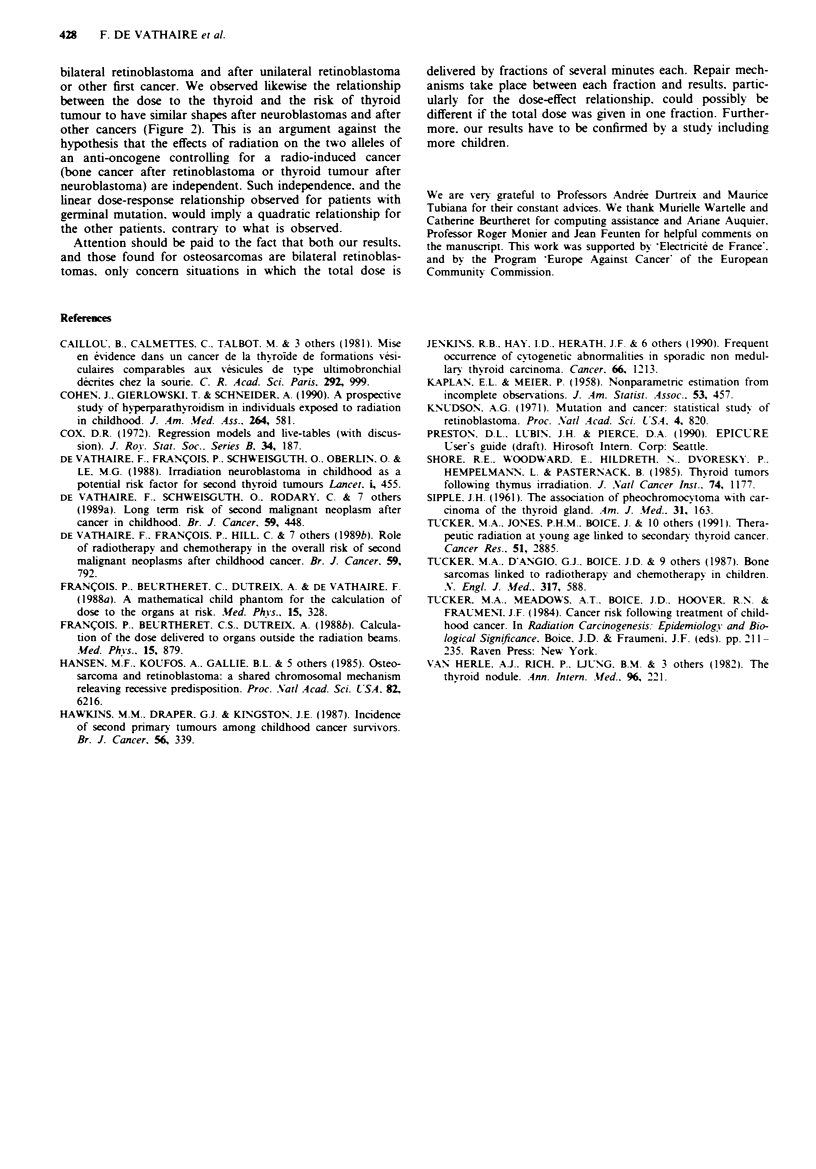# Epidemiological evidence for a common mechanism for neuroblastoma and differentiated thyroid tumour.

**DOI:** 10.1038/bjc.1992.87

**Published:** 1992-03

**Authors:** F. de Vathaire, P. François, M. Schlumberger, O. Schweisguth, C. Hardiman, E. Grimaud, O. Oberlin, C. Hill, J. Lemerle, R. Flamant

**Affiliations:** Unité de Recherche en Epidémiologie des Cancers (U287 INSERM), Villejuif, France.

## Abstract

Because genetic predisposition probably plays an important role in the aetiology of most of childhood cancers, studies of second primaries occurring after these cancers may be particularly informative about possible common genetic mechanisms in both of these cancers. We have studied the incidence of thyroid tumours occurring after cancer in childhood in a cohort of 592 children treated before 1970. Among these children, six later developed a thyroid carcinoma, and 18 developed a thyroid adenoma. Radiation doses received to the thyroid by each of the irradiated children have been estimated using individual radiotherapeutic technical records. Thyroid carcinomas and thyroid adenomas were five times more frequent after irradiation for neuroblastoma than after irradiation for any other first cancer. This ratio did not depend on sex, nor on time elapsed since irradiation, nor on dose of radiation received for the thyroid gland. This result suggests that there is a common mechanism for the occurrence of neuroblastoma and of differentiated thyroid tumour.


Br. J. Cancer (1992), 65, 425-428 © Macmillan Press Ltd., 1992

### Epidemiological evidence for a common mechanism for neuroblastoma and differentiated thyroid tumour

F. de Vathaire[1], P. François[2], M. Schlumberger[3], O. Schweisguth[4], C. Hardiman[1,2], E. Grimaud[1], O. Oberlin[4], C. Hill[1], J. Lemerle[4] & R. Flamant[5]

[1]Unité de Recherche en Epidémiologie des Cancers (U287 INSERM), [2]Service de Radiophysique, [3]Service de Médecine Nucléaire, [4]Service de Pédiatrie, [5]Direction, Institut Gustave Roussy, rue Camilles Desmoulins, 94805 Villejuif, Cedex, France.

**Summary** Because genetic predisposition probably plays an important role in the aetiology of most of childhood cancers, studies of second primaries occurring after these cancers may be particularly informative about possible common genetic mechanisms in both of these cancers. We have studied the incidence of thyroid tumours occurring after cancer in childhood in a cohort of 592 children treated before 1970. Among these children, six later developed a thyroid carcinoma, and 18 developed a thyroid adenoma. Radiation doses received to the thyroid by each of the irradiated children have been estimated using individual radiotherapeutic technical records. Thyroid carcinomas and thyroid adenomas were five times more frequent after irradiation for neuroblastoma than after irradiation for any other first cancer. This ratio did not depend on sex, nor on time elapsed since irradiation, nor on dose of radiation received for the thyroid gland. This result suggests that there is a common mechanism for the occurrence of neuroblastoma and of differentiated thyroid tumour.

Epidemiological studies of second primary cancers may be of great help in improving the knowledge of carcinogenesis, because evidence for association between two types of cancer may lead to the formulation of hypotheses concerning common mechanisms in both of these cancers. The observation of an excess incidence of osteosarcoma after bilateral retinoblastoma has led to the suggestion that the anti-oncogene Rb, which controls for retinoblastoma, played a role in the development of osteosarcoma, and this role has been demonstrated later (Knudson, 1971; Hansen *et al.*, 1985).

If such an association between two cancers is less strong, a cohort study including several types of first cancers needs to be organised in order to identify this association and to be able to adjust for the carcinogenic effects of the first cancer treatment.

We report here the results of a cohort study monitored in order to analyse the risk of thyroid tumour after a first cancer in childhood.

### Methods

### *Patients*

This study included all the 592 children treated for a cancer at the Gustave-Roussy Institute (IGR) between 1942 and 1969 and who were alive and free of disease 5 years after diagnosis, excluding 22 children treated for a thyroid cancer and 20 who had received brachytherapy. Those latter 20 children were excluded in order to include only patients treated with the same type of radiation at a similar dose rate. The diagnosis of cancer was confirmed by histology, cytology and measurements of tumour marker levels, or clinically when a tumour sample was not available, notably for brain tumours. The absence of children treated for leukaemia (Table I) is due to the fact that IGR is an important reference centre in France for solid paediatric cancers. All children were followed-up by one of us (O.S.). Only clinically apparent tumours of the thyroid gland were recorded.

Correspondence: F. de Vathaire.
Received 3 September 1991; and in revised form 11 November 1991.

### *Dosimetry*

The dose and duration of each drug received by the 592 children, and information on radiotherapy were extracted from medical records (Table I). Of the 592 children, 496 received X or gamma radiation. For each of these patients, the radiation doses to the two lobes of the thyroid gland were estimated retrospectively using individual radiotherapeutic technical records. The estimation involved two steps. The first step estimated the position of the two lobes of the thyroid gland from the size of the child at time of treatment, using a child phantom model based on auxometrical curves (Francois *et al.*, 1989*a*). The second step used a model which describes the variation of the dose in and outside the treatment beam, taking into account the quality of the radiation, the characteristics of the treatment machine (collimator and machine head construction) and the field shape (Francois *et al.*, 1989b). For each of the children in the study, data for this second step were obtained from the technical reports and from the controls films.

### *Statistical methods*

The cumulative incidence of thyroid tumour was estimated using the Kaplan-Meier method (Kaplan & Meier, 1958).

The analysis of risk for given categories was performed using the Cox's regression model (Cox, 1972). The relationship between the dose of radiation received to the thyroid and the risk of thyroid tumour was studied using Poisson regression model of the excess of relative risk of thyroid tumour as a linear function of the dose, and a multiplicative function of other factors. This model was fitted using Epicure software (Preston *et al.*, 1990).

### Results

At the end point of the study, 1 January 1986, 152 patients (26%) were lost to follow-up 80 (14%) before January 1982. The median follow-up from diagnosis of first cancer was 22 years (range: 5 to 40).

None of the 96 non-irradiated patients developed a thyroid tumour. Of the 496 irradiated patients, 24 developed an epithelial thyroid tumour. This leads to a cumulative incidence of thyroid tumour, 25 years after irradiation, equal to 8.1%, with a 95% confidence interval (CI) of 4.7-13.8%. Of

**Table I** Age, sex, and modalities for treatment of 592 children treated at Gustave Roussy Institute for a first cancer, and alive and free of disease 5 years after diagnosis

| | | | Radiotherapy | | | | | | | | Chemotherapy | |
|---|---|---|---|---|---|---|---|---|---|---|---|---|
| *First cancer (n)* | *Males (%)* | *Median age at first cancer (range)* | *n* | *Type of energy** *LEXR (n)* | *COB (n)* | *HEXR (n)* | *e⁻ (n)* | *Median dose at thyroid (cGy)* | *Median number of fractions* | *Median duration (days)* | *Any type (n)* | *Alkylating agents (n)* |
| All types (592) | 53 | 3 (0–17) | 496 | 246 | 166 | 100 | 36 | 50 | 18 | 36 | 303 | 122 |
| Neuroblastoma (99) | 54 | 0 (0–16) | 75 | 43 | 14 | 10 | 13 | 52 | 12 | 21 | 65 | 62 |
| Other (493) | 52 | 4 (0–17) | 421 | 203 | 152 | 90 | 23 | 50 | 19 | 38 | 238 | 60 |
| Wilm's tumour (175) | 51 | 2 (0–14) | 165 | 103 | 27 | 51 | 2 | 39 | 15 | 38 | 126 | 1 |
| Hodgkin's disease (38) | 63 | 10 (2–14) | 36 | 2 | 36 | 2 | 3 | 1533 | 18 | 37 | 28 | 19 |
| Brain tumour (80) | 45 | 7 (0–17) | 80 | 44 | 17 | 28 | 1 | 93 | 21 | 43 | 12 | 1 |
| Non-Hodgkin's lymphoma (32) | 59 | 7 (0–14) | 27 | 9 | 18 | 1 | 4 | 173 | 18 | 35 | 14 | 13 |
| Bone sarcoma (39) | 67 | 9 (0–14) | 29 | 4 | 26 | 0 | 1 | 17 | 24 | 42 | 10 | 8 |
| Soft tissue sarcoma (62) | 45 | 3 (0–14) | 40 | 19 | 12 | 1 | 4 | 22 | 18 | 32 | 23 | 4 |
| Other cancer (67) | 52 | 2 (0–14) | 44 | 22 | 16 | 6 | 5 | 47 | 19 | 37 | 25 | 14 |

*Some patients have received more than one type of energy. LEXR = low energy X-rays; COB = cobalt 60; HEXR = High energy X-rays; $e^-$ = electrons.

these 24 thyroid tumours, six were differentiated thyroid carcinomas and 18 were adenomas; this ratio of benign to malignant is similar to what has been reported in irradiated patients (Shore *et al.*, 1985) by other authors. We shall consider thyroid adenomas and carcinomas together in the rest of the study because of the small number of thyroid tumours observed and because the dose-response relationship between radiation and the risk of thyroid tumours has been found to be similar for adenomas and carcinomas by Shore *et al.* (1985) and of the same magnitude by Tucker *et al.* (1990).

Thyroid tumours were much more frequent after irradiation for neuroblastoma, 11 of 75 patients, than after irradiation for other cancer, 13 of 421 patients. This higher incidence of thyroid tumours after neuroblastoma was observed during the 30 years of follow-up (Figure 1), and existed both for carcinomas (3/75 *vs* 3/421) and for adenomas (8/75 *vs* 10/421).

When taking into account the other factors, no effect of age at irradiation was found (RR for children aged less than 2 years compared with others: 0.93):8/53 (15%) of the neuroblastoma patients treated under age of 2 years developed a thyroid tumour compared with 3/22 (12%) in the

**Figure 1** Cumulative incidence rate of thyroid tumour and 95% confidence interval (95% CI), by type of first cancer, in a population of 496 patients irradiated for a first cancer in childhood.

older patients, these proportions being respectively 3% and 3% for non-neuroblastoma.

No effect of chemotherapy, of any type of alkylating agent or of any other drug was found.

Controlling for possible differences in sex, time elapsed since irradiation (20 or >years) and radiation dose to the thyroid (in cGy), the risk of developing a thyroid tumour was found to be 5.0 times higher (95% CI: 3.3–7.5) for children irradiated for a neuroblastoma than for other children. Table II presents the risk of thyroid tumour as a function of the dose, for neuroblastoma and for other tumours.

When fitting the excess of relative risk of thyroid tumour as a linear function of the dose of radiation received to the thyroid and as a multiplicative function of sex, type of first cancer (neuroblastoma or not), and follow-up, no interaction was found between effect of sex or neuroblastoma and that of dose: the same linear coefficient for the dose was found whatever the sex and the type of first cancer. This result implies that women and children treated for neuroblastoma would have a more important spontaneous rate of thyroid tumour rather than a more important radiosensibility of the thyroid. Figure 2 presents the relative risks in Table II, and those predicted from a linear relationship using the same coefficient for neuroblastoma and other tumours.

As previously reported (de Vathaire *et al.*, 1988, 1989*a*,*b*), excluding thyroid tumours, no more second tumours were found after irradiation for a neuroblastoma (three of 75) than for other type of first cancer (24 of 421) (RR = 0.80). Contrary to other authors (Cohen *et al.*, 1990), we did not find any parathyroid adenoma associated with the thyroid tumours.

### Discussion

Our results were not affected by the important proportion of subjects lost to follow-up (22%): under the extreme assumption, more probable in France, that all children lost to follow-up were alive and free of thyroid tumour at the end of 1985, the relative risk of thyroid tumour was 4.2 (95% CI: 1.9–9.5) for neuroblastomas, compared with that for other tumours.

Tucker *et al.* (1991) suggested an excess of thyroid cancers after dactinomycin. We not confirm this result.

The two other studies of second thyroid tumours after cancer treatment considered only thyroid carcinomas and not adenomas. The Late Effect Study Group (Tucker *et al.*, 1984; 1991), a hospital-based study, observed seven second thyroid cancers among the 790 patients irradiated for neuroblastoma, compared with 16 among the 8,380 others. When the dose received to the thyroid was not taken into account, the risk of thyroid cancer was found to be 7.7 times higher after

**Table II** Risk of thyroid tumour according to the type of first cancer and to the dose of radiotherapy received at the thyroid gland

| *First cancer type* | *Radiotherapy dose to the thyroid gland in cGy (median dose)* 0 (no radiotherapy) | 0.1–50 (24) | 51–100 (67) | 101–500 (157) | 501–2000 (1299) | 2001–4192 (2419) |
|---|---|---|---|---|---|---|
| Neuroblastomas | | | | | | |
| Thyroid tumours/number of patients | 0/24 | 2/37 | 1/12 | 2/15 | 4/8 | 2/3 |
| Mean annual incidence of thyroid tumour per $10^4$ persons[b] | 0 | 46 | 54 | 86 | 336 | 444 |
| Risk of thyroid tumour relative to the reference category (95% CI)[c] | – | 6.6 (1.1–40) | 5.2 (0.5–50) | 9.5 (2–59) | 26 (6–119) | 58 (9–357) |
| Non-neuroblastomas | | | | | | |
| Thyroid tumours/number of patients | 0/71 | 3/124 | 1/61 | 2/58 | 5/71 | 2/17 |
| Mean annual incidence of thyroid tumour per $10^4$ persons[b] | 0 | 7.2 | 7.8 | 21 | 54 | 106 |
| Risk of thyroid tumour relative to the reference category (95% CI)[c] | – | 1[a] | 0.9 (0.2–12) | 1.5 (0.3–9) | 7.2 (1.7–30) | 20 (3.3–125) |

[a]Reference category; [b]The first 5 years following irradiation were excluded. [c]The relative risk of thyroid tumour is the ratio between the risk for the dose range considered and the risk in the reference category, namely non-neuroblastoma having received between 0.1 and 50 cGY. All relative risks were adjusted on sex and were estimated through Cox's regression model.

**Figure 2** Relative risk of thyroid tumour as a function of the radiation dose, by type of first cancer, logarithmic scale. The symbols represent, in a log-log scale, the relative risk of thyroid tumour (given in Table II) as a function of the dose to thyroid (□) = the risk for children treated for a neuroblastoma, * = the risk for children treated for another type of cancer. The curves correspond to Poisson regression models of the excess of relative risk of thyroid tumour as a linear function of the dose. For non-neuroblastomas, the model is: relative risk = 0.0055*dose. For children treated for a neuroblastoma, the model is: relative risk = 5.0 + 0.0055* dose. Where the dose is expressed in cGy and the risk is expressed relatively to a risk for a zero dose to the thyroid. The addition of a quadratic function of the dose or of an interaction term between the dose and the type of first cancer (non-parallelism of the two dose-response relationships), did not significantly improve the fit of the model (respectively, $\chi^2 = 0.1$; $P = 0.8$, and $\chi^2 = 0.1$; $P = 0.8$).

treatment for neuroblastoma than for other types of first cancer. When the dose received to the thyroid was taken into account, the authors found a rate of 2.1 thyroid cancers per person-years cGY after neuroblastoma, 1.6 after Wilms' tumour, 0.3 after Hodgkin's disease, and 0.3 after non-Hodgkin's lymphoma. Nevertheless, they were not able to control directly for differences in the dose to the thyroid between the different types of first cancers; the estimation of the dose of radiation to the thyroid was conducted on cases of thyroid cancer and on controls matched for type of first cancer. Hence, the estimations of rates per person-years cGy for the cohort seem to have been made by extrapolating the mean doses obtained for the controls and the cases in the case-control study to the entire cohort, and are imprecise. In a British registry-based cohort study (Hawkins *et al.*, 1987) involving more than 10,000 3-year survivors of childhood cancer, only three thyroid cancers were observed. No thyroid cancers were observed after a median follow-up of 13. 5 years among the 134 irradiated neuroblastomas. As a rule, more second cancers, and particularly more thyroid cancers, are found in hospital based-studies than in registry-based studies because of differences in treatment intensity and in extent of diagnostic investigations during follow-up. This difference in frequency is strongly increased for thyroid adenomas, whose diagnosis is difficult, and depends on the importance of nuclear medicine units, that have been particularly developed at IGR.

There are biological arguments supporting a relationship between thyroid tumours and neuroblastomas. Firstly, the tumours of the sympathetic system (pheochromocytomas and, in rare instances, neuroblastomas) can be associated with medullary thyroid carcinoma (Sipple, 1961). Secondly, there is evidence that C cells and epithelial thyroid cells share a common embryological origin (Caillou *et al.*, 1981). Thirdly, similar chromosomal deletions or abnormalities have been found in medullary thyroid carcinomas and in epithelial thyroid tumours (Jenkins *et al.*, 1990).

The higher incidence of thyroid cancer after neuroblastoma suggests a common mechanism for the occurrence of neuroblastoma and of differentiated thyroid tumour. This would imply an increased risk of thyroid tumour among non-irradiated neuroblastoma patients. Our series includes only 24 such children; even with a relative risk of 7.5 (upper level of the 95% CI of our estimation of 5.0) and with a ratio of 10 adenomas for one carcinoma (Van Herle *et al.*, 1982). The expected number of thyroid tumours among these 24 children, based on incidence rates from the national registry of Denmark (there is no national cancer incidence registry in France), is 0.39 and the probability of observing no thyroid tumour is 67%.

We observe an excess risk of thyroid cancer after neuroblastoma which could be compared with the excess of osteosarcoma after bilateral retinoblastoma. Nevertheless, the mechanism of the association between thyroid tumour and neuroblastoma is probably different for two reasons. First, neuroblastoma does not present in a familial form. Second, bilateral or generalised neuroblastomas do not provide a higher risk of thyroid tumour: among the 75 irradiated neuroblastoma patients in our series, three were bilateral and 12 were generalised, of which none developed a thyroid tumour.

Tucker *et al.* (1987) found the relationships between the dose of radiation received by a given bone and the risk of osteosarcoma in this bone, to be of similar shape after bilateral retinoblastoma and after unilateral retinoblastoma or other first cancer. We observed likewise the relationship between the dose to the thyroid and the risk of thyroid tumour to have similar shapes after neuroblastomas and after other cancers (Figure 2). This is an argument against the hypothesis that the effects of radiation on the two alleles of an anti-oncogene controlling for a radio-induced cancer (bone cancer after retinoblastoma or thyroid tumour after neuroblastoma) are independent. Such independence, and the linear dose-response relationship observed for patients with germinal mutation, would imply a quadratic relationship for the other patients, contrary to what is observed.

Attention should be paid to the fact that both our results, and those found for osteosarcomas are bilateral retinoblastomas, only concern situations in which the total dose is delivered by fractions of several minutes each. Repair mechanisms take place between each fraction and results, particularly for the dose-effect relationship, could possibly be different if the total dose was given in one fraction. Furthermore, our results have to be confirmed by a study including more children.

We are very grateful to Professors Andrée Durtreix and Maurice Tubiana for their constant advices. We thank Murielle Wartelle and Catherine Beurtheret for computing assistance and Ariane Auquier, Professor Roger Monier and Jean Feunten for helpful comments on the manuscript. This work was supported by 'Electricité de France', and by the Program 'Europe Against Cancer' of the European Community Commission.

### References

CAILLOU, B., CALMETTES, C., TALBOT, M. & 3 others (1981). Mise en évidence dans un cancer de la thyroïde de formations vésiculaires comparables aux vésicules de type ultimobronchial décrites chez la sourie. *C. R. Acad. Sci. Paris*, **292**, 999.

COHEN, J., GIERLOWSKI, T. & SCHNEIDER, A. (1990). A prospective study of hyperparathyroidism in individuals exposed to radiation in childhood. *J. Am. Med. Ass.*, **264**, 581.

COX, D.R. (1972). Regression models and live-tables (with discussion). *J. Roy. Stat. Soc., Series B*, **34**, 187.

DE VATHAIRE, F., FRANÇOIS, P., SCHWEISGUTH, O., OBERLIN, O. & LE, M.G. (1988). Irradiation neuroblastoma in childhood as a potential risk factor for second thyroid tumours *Lancet*, **i**, 455.

DE VATHAIRE, F., SCHWEISGUTH, O., RODARY, C. & 7 others (1989a). Long term risk of second malignant neoplasm after cancer in childhood. *Br. J. Cancer*, **59**, 448.

DE VATHAIRE, F., FRANÇOIS, P., HILL, C. & 7 others (1989*b*). Role of radiotherapy and chemotherapy in the overall risk of second malignant neoplasms after childhood cancer. *Br. J. Cancer*, **59**, 792.

FRANÇOIS, P., BEURTHERET, C., DUTREIX, A. & DE VATHAIRE, F. (1988*a*). A mathematical child phantom for the calculation of dose to the organs at risk. *Med. Phys.*, **15**, 328.

FRANÇOIS, P., BEURTHERET, C.S., DUTREIX, A. (1988*b*). Calculation of the dose delivered to organs outside the radiation beams. *Med. Phys.*, **15**, 879.

HANSEN, M.F., KOUFOS, A., GALLIE, B.L. & 5 others (1985). Osteosarcoma and retinoblastoma: a shared chromosomal mechanism releaving recessive predisposition. *Proc. Natl Acad. Sci. USA*, **82**, 6216.

HAWKINS, M.M., DRAPER, G.J. & KINGSTON, J.E. (1987). Incidence of second primary tumours among childhood cancer survivors. *Br. J. Cancer*, **56**, 339.

JENKINS, R.B., HAY, I.D., HERATH, J.F. & 6 others (1990). Frequent occurrence of cytogenetic abnormalities in sporadic non medullary thyroid carcinoma. *Cancer*, **66**, 1213.

KAPLAN, E.L. & MEIER, P. (1958). Nonparametric estimation from incomplete observations. *J. Am. Statist. Assoc.*, **53**, 457.

KNUDSON, A.G. (1971). Mutation and cancer: statistical study of retinoblastoma. *Proc. Natl Acad. Sci. USA*, **4**, 820.

PRESTON, D.L., LUBIN, J.H. & PIERCE, D.A. (1990). EPICURE User's guide (draft). Hirosoft Intern. Corp: Seattle.

SHORE, R.E., WOODWARD, E., HILDRETH, N., DVORESKY, P., HEMPELMANN, L. & PASTERNACK, B. (1985). Thyroid tumors following thymus irradiation. *J. Natl Cancer Inst.*, **74**, 1177.

SIPPLE, J.H. (1961). The association of pheochromocytoma with carcinoma of the thyroid gland. *Am. J. Med.*, **31**, 163.

TUCKER, M.A., JONES, P.H.M., BOICE, J. & 10 others (1991). Therapeutic radiation at young age linked to secondary thyroid cancer. *Cancer Res.*, **51**, 2885.

TUCKER, M.A., D'ANGIO, G.J., BOICE, J.D. & 9 others (1987). Bone sarcomas linked to radiotherapy and chemotherapy in children. *N. Engl. J. Med.*, **317**, 588.

TUCKER, M.A., MEADOWS, A.T., BOICE, J.D., HOOVER, R.N. & FRAUMENI, J.F. (1984). Cancer risk following treatment of childhood cancer. In *Radiation Carcinogenesis: Epidemiology and Biological Significance*. Boice, J.D. & Fraumeni, J.F. (eds). pp. 211–235. Raven Press: New York.

VAN HERLE, A.J., RICH, P., LJUNG, B.M. & 3 others (1982). The thyroid nodule. *Ann. Intern. Med.*, **96**, 221.